# Self-Sensing NiFe@N-doped Carbon Aerogel: Integrating Excellent Radar Stealth, Inherent Structural Health Monitoring, Thermal Management, and Flame Retardancy

**DOI:** 10.1007/s40820-026-02128-5

**Published:** 2026-03-10

**Authors:** Xiaosen Du, Jianhua Zhou, Jiarui Yu, Xiaoyan Nie, Mingyu Luo, Xingyuan He, Anguo Xiao

**Affiliations:** 1 College of Bioresources Chemical and Materials Engineering, Shaanxi University of Science and Technology, Xi’an, 710021 People’s Republic of China; 2 Hunan Provincial Key Laboratory of Water Treatment Functional Materials, Hunan University of Arts and Science, Changde, 415000 People’s Republic of China; 3 National Demonstration Center for Experimental Light Chemistry Engineering Education (Shaanxi, University of Science and Technology), Xi’an, 710021 People’s Republic of China

**Keywords:** Biomass-based carbon aerogel, Microwave attenuation, Simulation, Structural integrity monitoring, Multifunction

## Abstract

Biomass carbon-based aerogels derived from collagen protofibrils are gaining considerable attention in electromagnetic protection. However, achieving a well-designed microstructure, optimized magnetic and dielectric loss components, and integrated multifunctionality within a single material system remains a significant challenge. Herein, a three-dimensional (3D) hierarchically biomimetic honeycomb-like porous magnetic NiFe@N-doped carbon aerogel (NFNCA) is obtained via a simple strategy involving in situ growth, freeze-drying, and pyrolysis carbonization. Driven by the synergy of a 3D conductive networking structure, magnetic and dielectric multi-components, numerous heterogeneous interfaces, and diverse loss pathways, the optimized NFNCA exhibits exceptional electromagnetic wave attenuation capability, evidenced by a minimum reflection loss (*R*_L_) of −53.49 dB at 1.93 mm and an effective absorption bandwidth of 6.24 GHz (11.76–18.00 GHz). Furthermore, the exceptional radar stealth, infrared thermal stealth, thermal management, and flame retardancy characteristics of NFNCA render it a promising candidate for multiple applications in demanding environments. Interestingly, the 3D cross-linked conductive network of NFNCA can serve as strain sensors to detect changes in the internal structure of carbon aerogels. Hence, this work provides a feasible design strategy for developing lightweight, high-efficiency, and multifunctional biomass-based carbon aerogel electromagnetic wave absorbing materials for various application scenarios.

**Supplementary Information:**

The online version contains supplementary material available at 10.1007/s40820-026-02128-5.

## Highlights

- The biomimetic honeycomb-like porous magnetic NiFe@N-doped carbon aerogel (NFNCA) was efficiently fabricated through chemical cross-linking, in situ growth, unidirectional freeze-drying, and pyrolysis carbonization.
- The synergistic effect arising from the 3D conductive networking structure, diverse heterogeneous interfaces, magnetic/dielectric multi-component, and multiple loss pathways of NFNCA endowed this carbon aerogel with outstanding impedance matching and electromagnetic wave attenuation performance.
- The NFNCA featured excellent microwave attenuation, real-time monitoring of structural integrity, infrared thermal stealth, thermal management, and flame retardancy capabilities.

**Supplementary Information:**

The online version contains supplementary material available at 10.1007/s40820-026-02128-5.

## Graphical abstract

**Figure:**
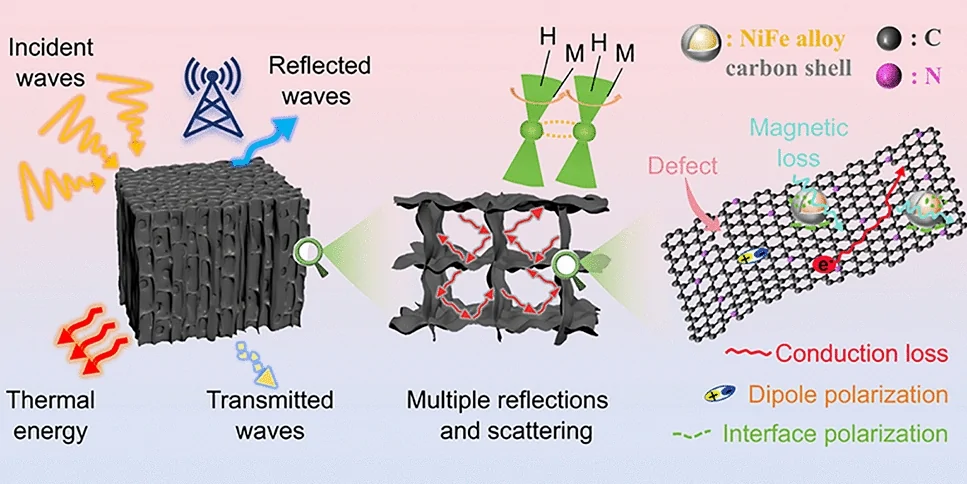

## Introduction

The rapid advancement of 5G technology has resulted in unprecedented societal progress and remarkable improvements in human life. Nevertheless, this development has heightened worries about electromagnetic wave (EMW) pollution and posed a potential threat to public health [1–5]. Hence, the design of materials and structures with high-efficiency EMW absorption has garnered considerable attention. To meet the practical demands of advanced electronics (e.g., 5G base stations, smart devices), aerospace, automotive industry, and smart buildings, ideal electromagnetic wave absorbing (EMWA) materials are required to possess not only the “thin, lightweight, broad, and strong” characteristics, but also superior environmental adaptability, such as excellent mechanical properties, thermal insulation, and flame retardancy [6]. Most importantly, real-time structural integrity monitoring has emerged as an indispensable criterion for promptly detecting unforeseen or subtle in-service damage, thereby maintaining the reliability of EMWA materials [7, 8]. Carbon-based materials, including graphene [9], carbon nanofibers [10], carbon nanocages/microspheres [11], and carbon aerogels, are regarded as extremely promising candidates for the development of outstanding EMWA materials. This is attributed to their inherent dielectric characteristics like transported electrons and dipoles, which are predominant in the internal electromagnetic response and energy conversion processes [12]. Moreover, carbon-based materials’ unique physical characteristics (e.g., well-developed pore structure, excellent thermal stability, and electrical properties) offer a promising pathway to meet the advanced requirements for environmental adaptability and structural health monitoring. However, synergistically integrating these multiple functionalities into a single, lightweight, and robust structure remains a significant challenge.

Recently, carbon aerogels derived from diverse biomass sources, such as wood [13, 14], cellulose [15–17], chitin [18, 19], and collagen protofibril [20], have attracted significant attention due to their environmental friendliness, widespread availability, abundant functional groups, large specific surface areas, and high porosity. Among these biomass sources, collagen protofibril extracted from leather industry wastes has emerged as an ideal choice, featuring high nitrogen content, numerous polar functional groups, excellent biodegradability and biocompatibility [21–23]. This sustainable strategy reduces reliance on petrochemical materials and mitigates industrial waste disposal pressure. Nitrogen doping can lead to diverse structural defects in the carbon skeleton and alter the distribution of the electron cloud, guiding the generation of electric dipoles under electromagnetic field conditions, which triggers dipole polarization and facilitates the attenuation of EMWs [24]. Although N-doped carbon aerogels derived from collagen protofibrils exhibit great potential for efficient EMW absorption, their fragile mechanical properties remain a primary challenge, limiting the application in complex practical environments. Cross-linking the collagen protofibril with various agents, such as glutaraldehyde [25], tannic acid [26], and metal ion [27], prior to heat treatment is a key strategy to fortify the network and thus enhance the mechanical properties of the final carbon aerogel. However, the EMW attenuation capability of modified collagen protofibril-based carbon aerogels still remains unsatisfactory due to the single dielectric loss mechanism. The synergistic electromagnetic loss effect of dielectric/magnetic hybrid materials can diversify loss mechanisms and optimize impedance matching, thereby enhancing the EMW attenuation capability [28, 29]. Magnetic components can be generated through carbonization of metal–organic frameworks (MOFs) [29, 30], which offer notable advantages including adjustable porosity, high dispersibility, easy component adjustability, and strong magnetization. In comparison with other MOFs, magnetic particles derived from Prussian blue analogues (PBAs) leverage extra advantages, such as higher metal content, readily tunable metal species, and smaller particle size, making them more conducive to enhancing magnetization and electromagnetic compatibility [31–33]. However, the conventional ex situ compositing method (mechanical blending) often results in poor interfacial compatibility between MOFs (including PBAs) and carbon matrix. This leads to a non-uniform distribution of the MOF-derived phases, generating agglomerated and isolated conductive islands that impair the EMW attenuation capability [34]. A more effective “bottom-up” approach, anchoring MOFs onto polymer backbones such as cellulose [35] and chitosan [36] prior to gelation, has been proven highly effective. However, to the best of our knowledge, no study has yet reported a strategy that utilizes in situ growth of MOFs within collagen protofibrils to fabricate high-performance, multifunctional magnetic carbon aerogels with desirable interfacial connectivity and uniform particle dispersion.

Herein, we reported an extensible strategy utilizing dialdehyde cellulose nanofibril (DCNF) as a cross-linking agent to fabricate biomimetic honeycomb-like porous magnetic NiFe@N-doped carbon aerogel (NFNCA) through simple in situ growth, unidirectional freeze-drying, and pyrolysis carbonization. Through a facile chemical cross-linking approach, the formation of Schiff base covalent bonds between DCNF and collagen protofibrils could promote the construction of a three-dimensional (3D) network structure, providing favorable conditions for establishing a robust carbon aerogel skeleton. The abundant functional groups (e.g., carboxyl, amino, hydroxyl, amide, and imine groups) within the DCNF-crosslinked collagen protofibril network endowed it with a unique capacity for interfacial interactions, enabling it to coordinate with metal ions to form robust interfacial bonds. The in situ growth of NiFe-PBA particles within the DCNF-crosslinked collagen protofibril network resulted in a magnetic carbon aerogel featuring low density, high magnetic nanoparticle dispersion, strong interfacial connectivity, and superior mechanical properties. Significantly, the electrical conductivity, magnetism, and EMW attenuation capability of the NFNCAs could be readily obtained by adjusting the loading content of NiFe-PBA particles. The optimized sample exhibited excellent EMW attenuation capability, with a minimum reflection loss (*R*_L_) of −53.49 dB achieved at 1.93 mm and a wide broad effective absorption bandwidth (EAB) of 6.24 GHz obtained at 1.60 mm. In addition, NFNCAs exhibited real-time structural integrity monitoring, exceptional infrared thermal stealth, thermal management, and flame retardancy capabilities. This work opens new avenues for future exploration of high-performance multifunctional biomass-based carbon aerogels and the NFNCA demonstrates significant prospects for application in diverse fields, such as next-generation electronics, specialized equipment protection, aerospace, and defense applications.

## Experimental Section

### Fabrication of DCNF

DCNFs rich in aldehyde functional groups were prepared by the selective oxidation of cellulose nanofibrils (CNFs) with NaIO_4_. The aldehyde content of DCNF was 3.48 ± 0.15 mmol g^−1^ as determined by the hydroxylamine hydrochloride/sodium hydroxide method. Detailed experimental processes are available in Sects.  1.2 and 1.3 of the Supporting Information.

### Fabrication of NiFe@N-doped Carbon Aerogel

**Fig. 1:**
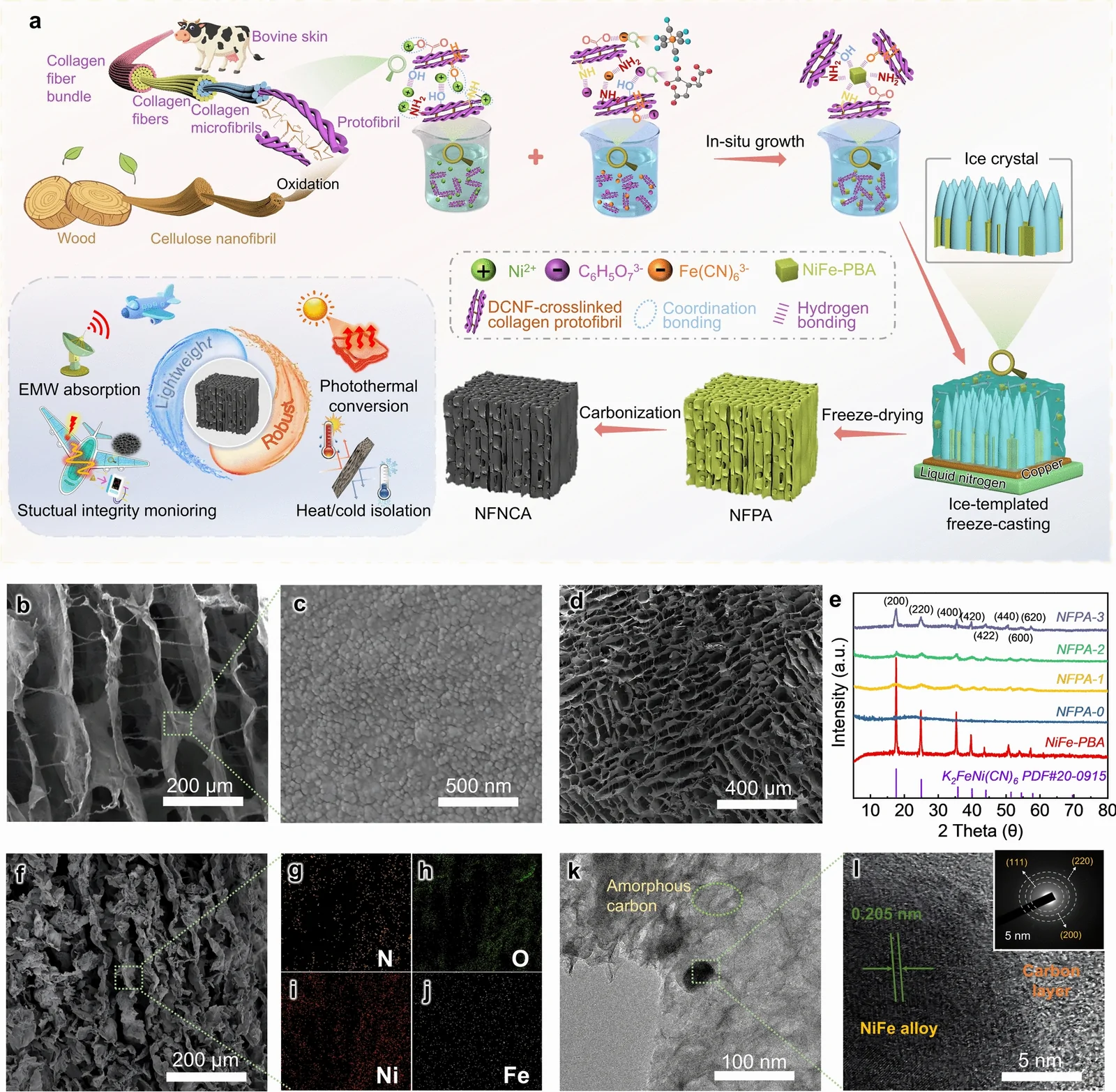
**a** Schematic diagram of fabricating NFNCA for multifunctional applications. **b** SEM image of NFPA-2 in the longitudinal section.** c** SEM image of in situ grown NiFe-PBA particles in NFPA-2. **d** SEM image of NFPA-2 in the transverse section. **e** XRD spectra of NiFe-PBA particles and NFPAs with different metal salt contents. **f** SEM image of NFNCA-2 in the longitudinal section. **g-j** EDS mapping images of N, O, Ni, and Fe elements. **k** TEM, **l** HR-TEM and selected area electron diffraction (SAED) of NFNCA-2

The preparation process of NiFe@N-doped carbon aerogel (NFNCA) is displayed in Fig. 1a. The DCNF content could affect the mechanical properties, density, and porosity of NFNCA. According to Fig. S1, when the DCNF content was 45 wt% of collagen protofibril, the comprehensive performance of NFNCA was optimal. Specifically, 0.2343 g of collagen protofibril and 0.1054 g of DCNF were dispersed in 25 mL of deionized water and stirred for 2 h to form mixture A. Then, 0.0776 g of Ni(NO_3_)_2_·6H_2_O and 0.1177 g of Na_3_C_6_H_5_O_7_·2H_2_O were dissolved in 5 mL of deionized water. The resulting solution was mixed with half of mixture A and subsequently magnetically stirred at 500 rpm for 15 min to yield suspension B. Similarly, 5 mL of deionized water was used to dissolve 0.0585 g of K_3_Fe(CN)_6_. The resultant solution was then mixed with the remaining mixture A under magnetic stirring at 500 rpm for 15 min to yield suspension C. Subsequently, suspension B was quickly poured into suspension C and magnetically stirred at 500 rpm for 15 min. Then, the resultant mixture was transferred into a specially designed Teflon mold with a copper base, followed by standing at room temperature for 24 h to enable the in situ growth of NiFe-PBA particles. To create a directional temperature differential, the mold was placed on the surface of a copper column immersed in liquid nitrogen for unidirectional freezing until the suspension was completely frozen. Subsequently, the NiFe-PBA/collagen protofibril composite aerogels (NFPAs) were produced via freeze-drying at −55 °C for 72 h. By adjusting the content of metal salts (27.2, 42.8, or 59.9 wt%), NFPAs with different NiFe-PBA contents were synthesized and designated as NFPA-1, NFPA-2, and NFPA-3, respectively. Finally, the NFPAs were put in a quartz boat and heated to 650 °C under an Ar atmosphere for 2 h with a heating rate of 5 °C min^−1^ to obtain NFNCAs, which were denoted as NFNCA-1, NFNCA-2, and NFNCA-3, respectively. For comparison, NFPA-0 without metal salts and its pyrolyzed product NFNCA-0 were prepared following the same procedure as described above.

## Results and Discussion

### Morphology and Structure

The fabrication process of NiFe@N-doped carbon aerogel involved the in situ synthesis of NiFe-PBA particles within a DCNF-crosslinked collagen protofibril network, following unidirectional freeze-drying and pyrolysis carbonization processes (Fig. 1a). Initially, the Schiff base reaction occurred between the amino groups of collagen protofibrils and the aldehyde groups of DCNF (Fig. S2), thereby generating a cross-linked network structure. The DCNF-crosslinked collagen protofibril network had a large number of carboxyl, amino, hydroxyl, amide, and imine groups, which made it possible for Ni^2+^ ions to interact with the network directly. Meanwhile, Ni^2+^ ions within the DCNF-crosslinked collagen protofibril network formed complexes with ferrocyanide ions, through which the NiFe-PBA nuclei were anchored onto the DCNF-crosslinked collagen protofibril network and continuously grew into NiFe-PBA particles. The formation of a coordination complex between the DCNF-crosslinked collagen protofibril network and NiFe-PBA particles could be confirmed by the blue shift of the characteristic peak corresponding to the associated hydroxyl groups (-OH) in the Fourier transform infrared (FTIR) spectra (Fig. S3). Finally, the rigid NiFe-PBA particles were firmly anchored within the DCNF-crosslinked collagen protofibril network via strong interfacial coordination bonds, forming a stable 3D cross-linked system. In addition, the zeta potential of composite dispersion increased progressively with the NiFe-PBA loading content (Fig. S4), indicating that in situ growth of NiFe-PBA particles enhanced colloidal stability. This drove the formation of the jelly-like gel (Fig. S5).

**Fig. 2:**
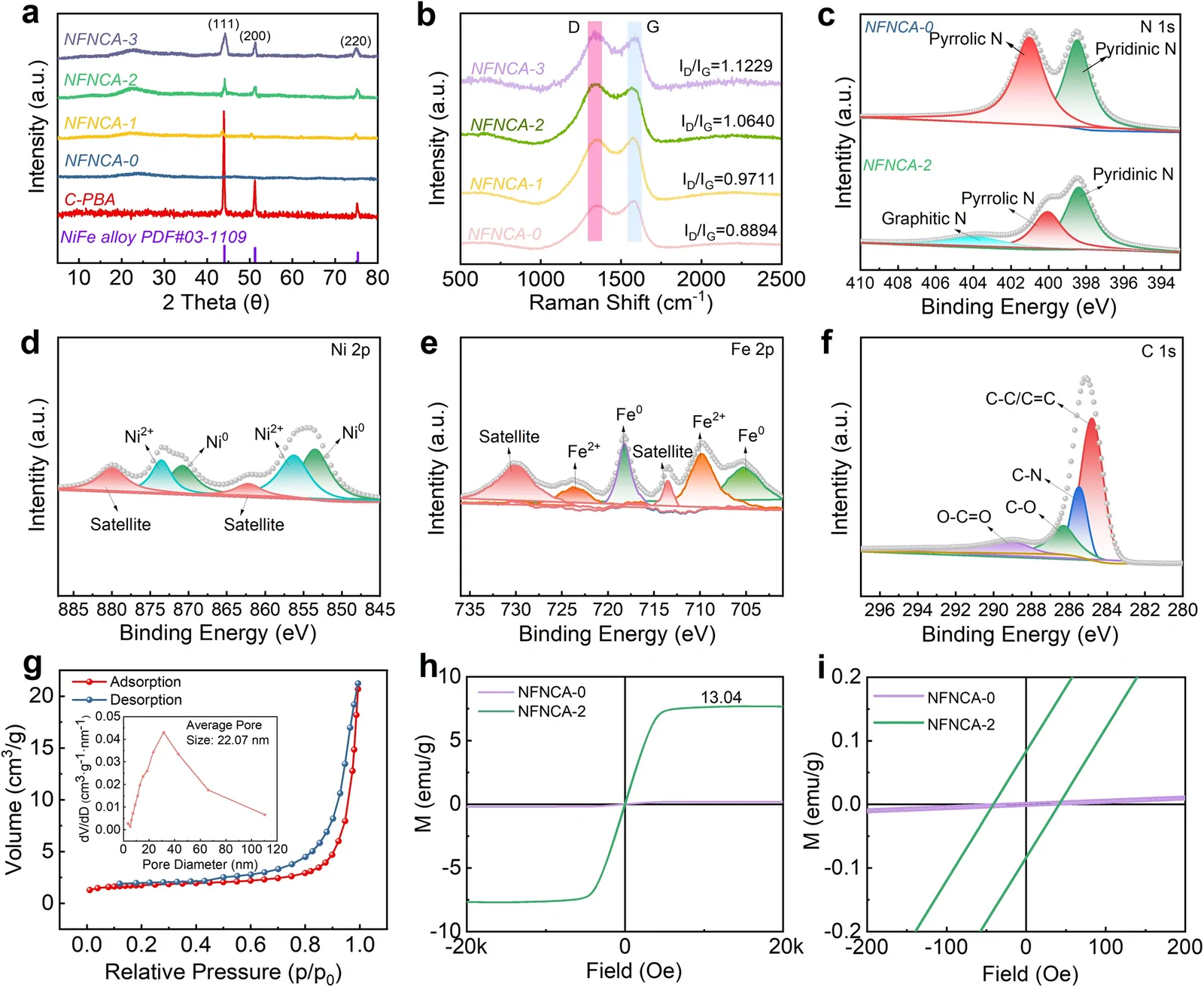
**a** XRD patterns of the NFNCAs and carbonized NiFe-PBA (C-PBA).** b** Raman spectra of the NFNCAs with different NiFe-PBA contents. **c** N 1*s* XPS spectra of NFNCA-0 and NFNCA-2. **d-f** Ni 2*p*, Fe 2*p*, and C 1*s* XPS spectra of NFNCA-2. **g** N_2_ adsorption–desorption isotherms of NFNCA-2. **h-i** Hysteresis lines and their local magnification diagram of NFNCA-0 and NFNCA-2

During the freezing phase, the unidirectional ice crystals acted as templates, and the NiFe-PBA/DCNF-crosslinked collagen protofibril hybrids was excluded by the growing ice crystals, allowing them to aggregate between ice crystals and form directional pore walls [37, 38]. Therefore, the NFPAs achieved an anisotropic pore structure after freeze-drying. The SEM image (Fig. 1b) revealed that NFPA possessed aligned cell walls, along with interconnected “bridges” between the parallel lamellae. NiFe-PBA particles existed in NFPA and were uniformly distributed (Fig. 1c). The surrounding matrix partially covered the NiFe-PBA particles, indicating that the nanocrystals were effectively embedded in the NFPA walls with good interfacial connectivity. Especially, NFPA presented a typical honeycomb-like biomimetic structure in transverse section (Fig. 1d). The X-ray diffraction (XRD) patterns of NiFe-PBA particles and NFPAs were consistent with those of standard pattern of K_2_FeNi(CN)_6_ (PDF#20–0915), which confirmed the successful synthesis of NiFe-PBA particles within NFPAs (Fig. 1e). In terms of morphology, after carbonization, the cell walls of the absorber became more curved and wrinkled, and the average pore gap decreased while the directional pore structure was reserved (Fig. 1f). Moreover, the energy-dispersive spectroscopy (EDS) element mapping indicated that N, O, Ni, and Fe elements in NFNCA-2 were well distributed (Fig. 1g-j), which implied that the in situ growth of NiFe-PBA on the carbon matrix was uniform. NiFe alloy nanoparticles with approximately sphere-like morphology and about 57 nm in diameter were observed to be embedded in the amorphous carbon matrix, as visualized by low-resolution transmission electron microscope (TEM) (Fig. 1k). Moreover, the high-resolution TEM image clearly revealed that the lattice spacing of the crystalline metal particles was 0.205 nm (Fig. 1l). Furthermore, the selected area electron diffraction (SAED) pattern (Fig. 1l inset) revealed the crystal planes (111), (200), and (220) of the NiFe alloy, which was also confirmed in XRD pattern (Fig. 2a) [39]. Overall, the embedding of these crystalline NiFe alloy nanoparticles within the carbon matrix generated abundant heterogeneous interfaces. These interfaces acted as polarization centers, facilitating charge accumulation and thereby significantly enhancing the EMW attenuation capability.

The crystalline structure of the NFNCAs was investigated by XRD, revealing three distinct diffraction peaks at 2θ = 44.14°, 51.28°, and 75.37° (Fig. 2a). These diffraction peaks, respectively, corresponded to the (111), (200), and (220) planes of the NiFe alloy (PDF#03–1109). Additionally, a broad diffraction peak was detected between 20° and 25°, indicating the existence of graphite carbon. The absence of impurity peaks indicated that NiFe@C nanoparticles possessed good crystallinity and stability, which stemmed from the protection provided by the graphite carbon shell. To analyze the lattice defects in the NFNCAs, Raman spectroscopy was utilized. The spectra of all samples obviously presented two distinct peaks: the D band (1350 cm^−1^) and the G band (1590 cm^−1^). The D band is caused by the disordered arrangement of carbon atoms in the carbon framework, while the G band originates from all carbon sites with *sp*^2^ hybridization, including graphitic and non-graphitic sites. The intensity ratio of D band to G band (*I*_D_/*I*_G_) is typically employed as a metric for evaluating the relative graphitization degree of carbon materials [40, 41]. As the NiFe-PBA loading increased, the *I*_*D*_/*I*_*G*_ value rose (Fig. 2b), implying the presence of smaller *sp*^2^ carbon nanocrystallites and more lattice defects, which was beneficial for dipole polarization. In this case, the fragmented graphite crystals led to a partial shift in electron migration's conduction mode to electron hopping, which consequently reduced the electrical conductivity (Fig. S6). This was because NiFe-PBA was abundant in N atoms. N atoms had a similar atomic structure to that of C atoms, which promoted the doping of N atoms into the carbon matrix. As a result, the doped N atoms impaired the ordered structure of the graphitic carbon, forming defective structures and disordered carbon [42]. Functioning as potent polarization centers, these induced defects significantly intensified dipole polarization, hence elevating the overall EMW attenuation capability [43].

The elemental composition, chemical bonds, and oxidation states of NFNCA-0 and NFNCA-2 were analyzed by X-ray photoelectron spectroscope (XPS) (Figs. 2c-f, S7, and S8). In the analysis of full-spectra, NFNCA-0 appeared characteristic peaks of C, O, and N elements, and NFNCA-2 showed typical peaks of C, O, N, Ni, and Fe elements (Figs. S7a and S8a). The N 1*s* spectrum of NFNCA-2 revealed three deconvoluted peaks, which were attributed to graphitic N, pyrrolic N, and pyridinic N at binding energies of 401.51, 400.06, and 398.40 eV, respectively. However, for NFNCA-0 without NiFe-PBA, only pyrrolic N (400.89 eV) and pyridinic N (398.40 eV) peaks could be observed (Fig. 2c). This difference was because NiFe-PBA contained numerous cyano (-CN) groups, and the NiFe alloys acted as catalysts to promote the graphitization recombination of -CN groups during high-temperature carbonization, forming *sp*^2^ hybridized graphitic N [44]. Furthermore, the relative peak areas were used to calculate the relative amounts of each nitrogen species in both samples (Table S1). In NFNCA-0, the proportions of pyridinic N and pyrrolic N were 46.51 and 53.49 wt%, respectively, while in NFNCA-2, those of pyridinic N, pyrrolic N, and graphitic N were 51.56, 33.57, and 14.87 wt%, respectively. Pyridinic N favors the generation of lattice defects, pyrrolic N facilitates dipole polarization, and graphitic N can reduce the electrical conductivity of the carbon matrix [45–47]. Therefore, the synergistic effect of these three nitrogen species doped in carbon matrix contributed to the EMW absorption properties of NFNCA-2. The high-resolution Ni 2*p* and Fe 2*p* spectra of NFNCAs were analyzed to determine the elemental valence states. In the Ni 2*p* spectrum (Fig. 2d), the peaks positioned at 853.46 and 870.76 eV were assigned to metallic Ni (Ni^0^), while those located at 856.25 and 873.55 eV were associated with oxidized Ni species (e.g., Ni^2+^). Similarly, the Fe 2*p* spectrum revealed the coexistence of metallic Fe (Fe^0^) at 705.36 and 718.46 eV, and oxidized Fe species at 709.92 and 723.52 eV (Fig. 2e). These results confirmed that Ni and Fe in NiFe-PBA were reduced to form alloy particles, which was consistent with the previously presented TEM and XRD results (Figs. 1k, l and 2a). Simultaneously, the oxidation states of the metal elements revealed that alloy particles experienced slight oxidation on their surfaces upon air exposure, a phenomenon that was very common in MOF-derived materials [31]. The C 1*s* spectra of NFNCA-0 and NFNCA-2 could be deconvoluted into four peaks located at 284.80, 285.52, 286.60, and 288.83 eV. These peaks corresponded to C–C/C = C, C–N, C–O, and O–C = O, indicating the existence of various carbon-containing functional groups in the carbon matrix (Figs. S7b and 2f). The O 1*s* XPS spectra of NFNCA-0 and NFNCA-2 were congruous (Figs. S7c and S8b), both revealing three oxygen species attributed to O–C (531.81 eV), C–O–C (533.34 eV), and adsorbed water molecules (536.33 eV).

Based on the IV-type N_2_ adsorption–desorption isotherm, some pores were found in NFNCA-2 (Fig. 2g). This pore structure was mainly attributed to the imperfection of the carbon lattice, which was quite common in biomass-derived amorphous carbon. Moreover, the unidirectional structure of the carbon aerogel also influenced the formation of pores. According to the Maxwell–Garnett theory (Eqs. S3 and S4) [35], the air introduced into the pores could reduce the effective dielectric constant, which improved the absorber’s impedance matching, thereby enhancing the EMW attenuation capability.

The magnetic properties of NFNCA-0 and NFNCA-2 were characterized by VSM, and the hysteresis lines are shown in Fig. 2h, i. Compared with NFNCA-0, NFNCA-2 exhibited an obvious symmetrical hysteresis loop, indicating that the introduction of NiFe-PBA particles endowed the absorber with good ferromagnetic behavior. The saturation magnetization strength (*M*_*S*_) and coercivity (*H*_*c*_) of NFNCA-2 were 13.04 emu g^−1^ and 41.11 Oe, respectively. Due to the presence of the non-magnetic carbon matrix, these values were lower than those of bulk NiFe alloys (about 87.3 emu g^−1^ and 353 Oe) [39]. The lower coercive force weakened the magnetic anisotropy and thereby caused the natural resonant frequency to shift toward lower frequencies, which was beneficial for the attenuation of EMWs in the low-to-medium frequency range (Eqs. S5-S7).

### EMW Absorption Performance of NFNCAs

**Fig. 3:**
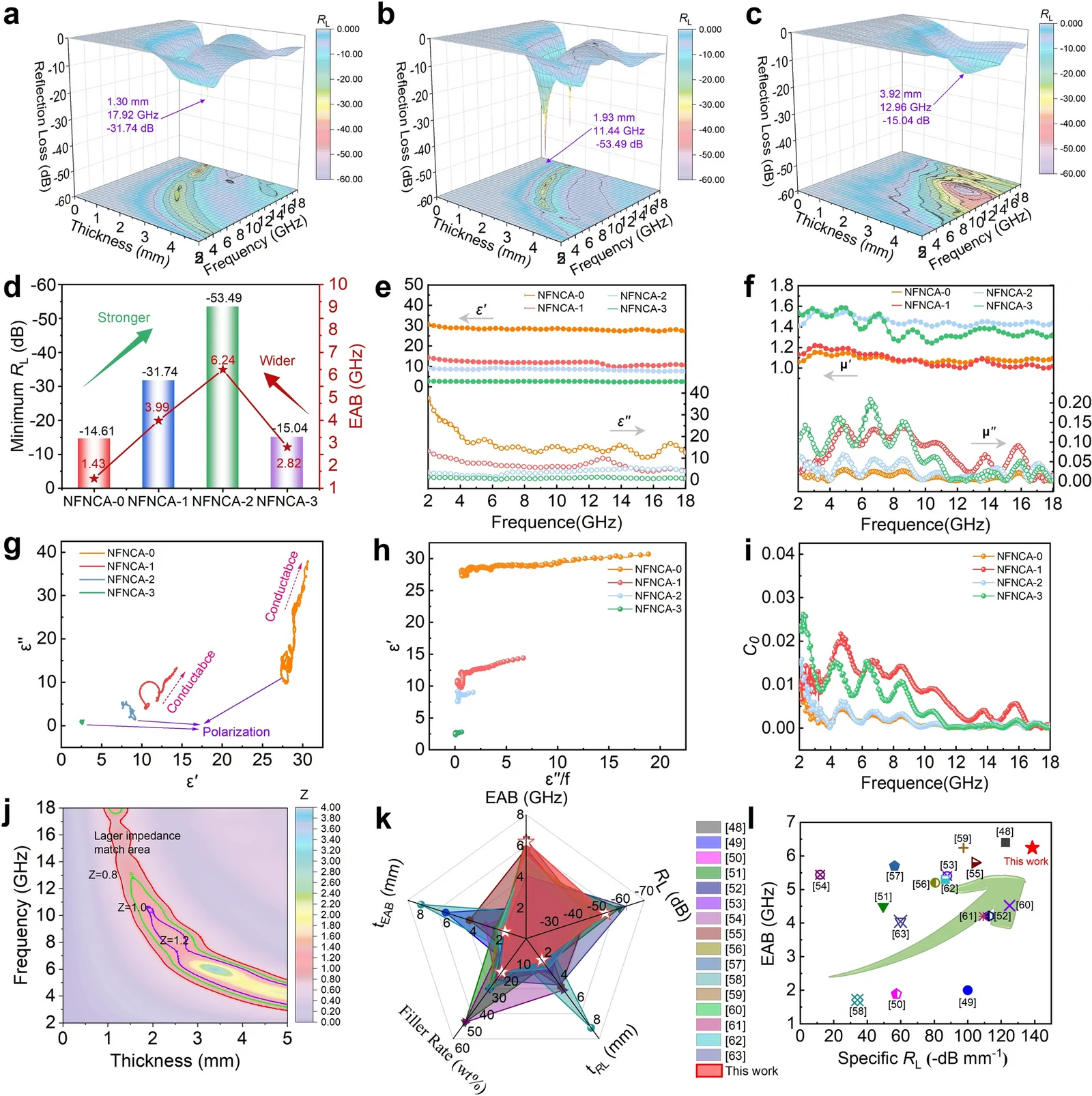
EMWA performance of **a** NFNCA-1, **b** NFNCA-2, and **c** NFNCA-3. **d** Comparison of the minimum *R*_L_ and EAB between samples. **e** *ε'* and *ε''* values for NFNCAs. **f** *μ'* and *μ''* values for NFNCAs. **g, h** Cole–Cole picture and the relationship between *ε'* versus *ε''*/*f* of NFNCAs. **i** Eddy current loss of NFNCAs. **j** Two-dimensional pattern of impedance values of NFNCA-2. **k** EMWA characteristics of NFNCA-2 compared with the reported carbon-based composites [48–63]. **l** Specific *R*_L_ value of NFNCA-2 compared with previously reported works

According to the transmission line theory (Eqs. S8 and S9), the widely used evaluation indicators for the EMW attenuation capability of absorbers include the *R*_L_ value and the EAB value. In general, an *R*_L_ value less than −10 dB indicates that more than 90% of the incident EMW energy is absorbed, and the corresponding frequency band with *R*_L_ < −10 dB is considered as the EAB. NFNCA-0 exhibited relatively weak EMW absorption capability, with a minimum *R*_L_ value of −14.61 dB (t = 0.88 mm, f = 15.55 GHz) and an EAB of 1.43 GHz (t = 0.88 mm, 14.81–16.24 GHz) (Fig. S9), which might be caused by its poor impedance matching characteristics (Fig. S16a, impedance matching diagram). In contrast, after introducing NiFe-PBA, the EMW attenuation capability of NFNCAs was significantly improved (Figs. 3a-c and S10-S12), indicating that the in situ grown NiFe-PBA had a positive effect on enhancing EMW absorption. Among them, NFNCA-1 achieved a minimum *R*_L_ value of −31.74 dB (t = 1.30 mm, f = 17.92 GH) and an EAB of 3.99 GHz (t = 1.49 mm, 14.00–17.99 GHz) (Figs. 3a and S10). Notably, the minimum *R*_L_ value of NFNCA-2 reached −53.49 dB (t = 1.93 mm, f = 11.44 GHz) (Figs. 3b and S11a). According to Eq. S10, NFNCA-2 could attenuate more than 99.99955% of EMW energy. Meanwhile, the EAB of NFNCA-2 reached 6.24 GHz (t = 1.60 mm, 11.76–18.00 GHz), covering the entire X-band (Fig. S11b). However, further increasing the NiFe-PBA loading content resulted in a decrease in EMW absorption performance. For instance, NFNCA-3 exhibited a minimum *R*_L_ value of −15.04 dB (t = 3.92 mm, f = 12.96 GHz) and an EAB of 2.82 GHz (t = 4.33 mm, 11.09–13.91 GHz) (Figs. 3c and S12), indicating that excessive NiFe alloy nanoparticles may hinder the construction of the 3D conductive network structure. NFNCA-2 demonstrated advantages in both absorption intensity and bandwidth (Fig. 3d), suggesting that a reasonable loading content of NiFe-PBA was crucial for achieving optimal EMW absorption capacity. Therefore, the EMW attenuation ability of NFNCAs could be regulated by tuning the loading content of NiFe-PBA. Furthermore, as thickness increased, the NFNCA-2 absorption frequency shifted toward lower frequencies, which could be described by the quarter-wavelength (1/4λ) cancellation theory (Eq. S11). The experimental *t*_*m*_ perfectly matched the theoretically estimated values, indicating the presence of interference-type loss in NFNCA-2 (Fig. S13) [64]. In summary, NFNCA-2 exhibited EMW absorption with selectivity of frequency. When the incident EMW frequency matched resonance frequency of the material, it would present maximum interference loss and optimal EMW attenuation capability.

To better explore the electromagnetic characteristics of NFNCAs, the dielectric constant (*ε*_*r*_ = *ε'*-j*ε''*) and permeability (*μ*_*r*_ = *μ'*-j*μ''*) were also studied. The real parts (*ε'*, *μ'*) denote the ability to storage electromagnetic energy, while the imaginary parts (*ε''*, *μ''*) represent the ability to dissipate electromagnetic energy [65]. The decrease in permittivity with increasing frequency (Fig. 3e), which indicates that the EMW attenuation process was mostly influenced by conductive losses [10]. Electromagnetic parameters (*ε'*, *ε"*, *μ'*, and *μ"*) of NFNCAs could be regulated by NiFe-PBA content. As compared with NFNCA-0, the introduction of NiFe-PBA particles reduced the complex permittivity. In contrast, the complex permeability increased as the NiFe-PBA content enhanced (Fig. 3f). However, the magnetic loss tangent (tan*δ*_*μ*_) remained significantly lower than the dielectric loss tangent (tan*δ*_*ε*_) across all loading levels (Fig. S14a, b), demonstrating that dielectric properties dominated EMW absorption.

To elucidate the underlying dielectric loss mechanisms, Cole–Cole analysis was performed. It could be clearly separated between contributions from conduction loss and polarization loss processes (Eq. S12). The presence of semicircles shows the polarization loss process, and the slope of the curve’s tail represents the conduction loss of samples. In the Cole–Cole plot (Fig. 3g), the upward tendency was not observed for NFNCAs with high NiFe-PBA loading content (NFNCA-2 and NFNCA-3), which indicated that NiFe alloy nanoparticles could inhibit the electrical conductivity (Fig. S6) [66]. A linear relationship between *ɛ'* and *ɛ*′′/*f* indicated that dipole polarization was the only dielectric loss process (Eq. S13). The relationship was nonlinear (Fig. 3h), which confirmed that both dipole and interfacial polarization processes existed in NFNCAs [67].

Furthermore, based on Debye theory, the imaginary part of permittivity is separated into two parts (*ε''* = *ε*_*c*_*''* + *ε*_*p*_*''*), with *ε*_*c*_*''* representing the conduction loss and *ε*_*p*_*''* representing the polarization loss (Eq. S14) [68]. The nonlinear least squares method was used to fit the conduction loss and polarization loss. Regardless of varying NiFe-PBA loading contents, the *ε*_*c*_*''* remained significantly larger than the *ε*_*p*_*''*, demonstrating that conduction loss played a dominant role in the overall dielectric loss (Fig. S15). This was because the NFNCAs formed a conductive network within the paraffin matrix, resulting in increased conductive loss, which facilitated the conversion of EMW energy into thermal energy [69]. Regarding *ε*_*p*_*''*, there existed multiple relaxations for NFNCAs. Specifically, *ε*_*p*_*''* mainly originated from polarization relaxation of functional groups and defect dipoles in NFNCAs. The enhancement of interfacial polarization resulted from the existence of unbalanced charge accumulation at the interface between the surface of NFNCA and NiFe-PBA, forming a capacitor-like structure, which was conducive to the dielectric polarization response [70]. Thus, the aforementioned-findings further confirmed the influence of three primary factors of interfacial polarization relaxation, defect dipole polarization relaxation, and conductive loss on the dielectric loss of the NFNCAs.

To elucidate the contribution of eddy current loss in the magnetic loss mechanism of NFNCAs, the *C*_0_ criterion (Eq. S15) was evaluated on the basis of the skin effect theory [71]. Theoretically, if magnetic loss is only caused by eddy current loss, the *C*_0_ values should remain constant across the measured frequency range. In contrast, the *C*_0_ values show notable fluctuations if magnetic loss is mostly driven by ferromagnetic resonance. The magnetic loss of NFNCAs consisted of natural resonance (2–10 GHz) and exchange resonance (10–18 GHz) (Fig. 3i). Meanwhile, the absence of flat regions in the *C*_0_ curves confirmed the absence of eddy current loss, which was mainly attributed to the tiny particle size of NiFe alloys [66].

Impedance matching (*Z*, Eq. S16) and attenuation constant (*α*, Eq. S17) are key factors for assessing EMW absorption capabilities. In general, impedance matching can be assessed using *Z* =|*Z*_in_/*Z*_0_|, and a* Z*-value within the range of 0.8–1.2 is regarded as exhibiting ideal impedance matching performance [72]. The *Z*-value graph of NFNCA-0 (Fig. S16a) showed that the impedance matching was poor, which was due to high electrical conductivity causing EMW reflection. In contrast, the introduction of NiFe-PBA and the formation of numerous heterogeneous interfaces in the carbon matrix led to the reduction of NFNCA electrical conductivity. Therefore, NFNCAs demonstrated enhanced impedance matching capabilities (Figs. 3j and S16b, c). Notably, NFNCA-2 exhibited the biggest ideal impedance matching region (Fig. 3j), suggesting that the employed strategy efficiently optimized impedance matching. Furthermore, the EMW attenuation capacity of EMWA materials is demonstrated by the α (Eq. S17) [73]. The value of α followed the order of NFNCA-0 > NFNCA-1 > NFNCA-2 > NFNCA-3 across the entire test frequency range (Fig. S17), which indicated that NFNCA-0 possessed the most effective EMW attenuation capability. Nevertheless, the excessive dielectric loss in NFNCA-0 induced the significant impedance mismatch, resulting in unsatisfactory EMW attenuation capability. In comparison with the EMWA materials in the previous literature (Table S2), NFNCA-2 demonstrated the wide EAB of 6.24 GHz, strong absorption of −53.49 dB and low filler rate (20 wt%), as shown in radar chart (Fig. 3k). Moreover, specific reflection loss (specific *R*_L_, calculated via Eq. S18) is usually used to explain the minimum *R*_L_ value *vs* thickness and filler rate, and can evaluate the material’s comprehensive advantages in terms of light weight, thin thickness, and efficient absorption properties [18]. While maintaining a wide EAB, the specific *R*_L_ value in this work reached −138.58 dB mm^−1^, which indicated that the EMW attenuation capability of the biomimetic honeycomb-like porous magnetic NFNCA was superior to that of most EMWA materials reported previously (Fig. 3l and Table S2).

### Electromagnetic Simulation Results and EMW Attenuation Mechanism of NFNCAs

**Fig. 4:**
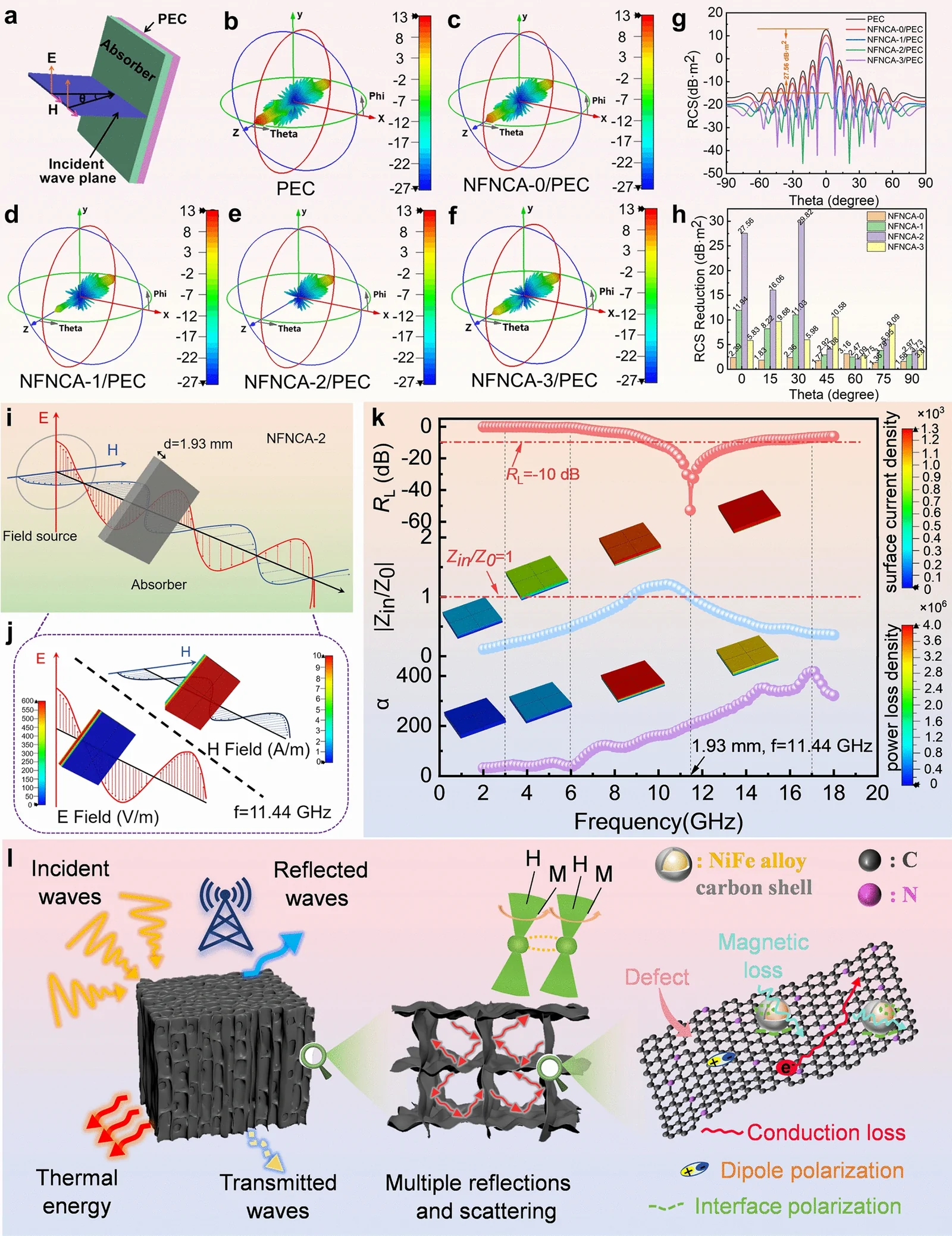
**a** Sketch of the CST simulation. CST simulation results of** b** PEC,** c** NFNCA-0/PEC, **d** NFNCA-1/PEC, **e** NFNCA-2/PEC, and **f** NFNCA-3/PEC.** g** PEC and NFNCAs/PEC RCS curves in the scattering angle range of 0° ± 90°. **h** RCS reduction values of NFNCAs/PEC.** i** COMSOL simulation model, **j** electric and magnetic field distribution at 11.44 GHz, and **k** comprehensive map of surface current density and power loss density. **l** Schematic diagram of EWA mechanisms for NFNCAs

To assess the EMW absorption capacity of NFNCAs minimum *R*_L_ actual far-field conditions, RCS simulations of perfect electric conductor (PEC) plates coated with NFNCA-0, NFNCA-1, NFNCA-2, and NFNCA-3 were performed using CST software (Fig. 4a-h). In this simulation model, the positive *Z* axis was defined as the direction of incidence, while theta represented the detection angle (Fig. 4a). Other simulation parameters were provided in Sect.  1.5 of the Supporting Information. The 3D simulated color maps and 2D RCS values of a PEC plate and PEC plates coated with NFNCAs between -90° to 90° are exhibited in Fig. 4b-f. The PEC plate exhibited the maximum radar scattering intensity, whereas PEC plates coated with NFNCAs showed significant reductions in radar scattering intensity, confirming the NFNCAs’ exceptional omnidirectional EMW absorption capability. The RCS values steadily dropped from 0° to ± 90° with several oscillations as the detection angle deviated (Fig. 4g). Compared to the PEC plate and other NFNCAs/PEC samples, NFNCA-2/PEC consistently exhibited the lowest RCS values (< −10 dB m^2^) throughout the −90° to 90° angular range, which corresponded well with its prominent EMW absorption performance (Fig. 3d). To corroborate the preceding statement, Fig. 4h shows a bar chart comparison of the RCS reduction values (the RCS values of PEC plate minus those of the samples). The NFNCA-2/PEC model exhibited the highest RCS reduction value of 29.82 dB m^2^ at 30°. The above results demonstrated that NFNCA-2 was effective in absorbing radar waves, resulting in excellent radar stealth capabilities.

The COMSOL finite-element method was employed to simulate the distribution of electric field intensity, magnetic field intensity, surface current density, and power loss density of NFNCA-2 (Fig. 4i). The distributions of the electric and magnetic fields at 11.44 GHz were illustrated in Fig. 4j, revealing that the electric field was primarily focused on the top, whereas the magnetic field was primarily distributed at the bottom region. Particularly, the power loss density distribution was consistent with the electric field distribution, which demonstrated that the attenuation effect of dielectric loss on EMWs was significant. The frequency at the minimum *R*_L_ point corresponded to the frequency at a high attenuation constant and Z = 1 (Fig. 4k). NFNCA-2 exhibited an outstanding EMW absorption capacity, confirming that simultaneous achievement of favorable impedance matching and strong attenuation was essential for superior EMW absorption performance. Surface current density and power loss density simulations of NFNCA-2 were conducted at frequencies of 3, 6, 11.44, and 17 GHz, respectively (Fig. 4k inset). Surface current density directly correlated with impedance matching characteristics, while power loss density reflected the attenuation capacity of the absorber (corresponding to α). Higher power loss density indicated enhanced EMWs energy dissipation. Evidently, the power loss density at 11.44 GHz exceeded those at 3, 6, and 17 GHz, which was in line with the evolving trend of EMW absorption performance (Figs. 3b and S11a). However, excessive surface current density at 17 GHz caused reflection, hindering the entry of EMWs and consequently reducing power loss density. This observation further substantiated that optimal impedance matching served as a critical prerequisite for achieving outstanding EMW attenuation capability.

Based on the above analysis, the schematic illustration of the proposed EMW attenuation mechanism of NFNCAs is presented in Fig. 4l. Firstly, the hierarchical porous structure of NFNCAs, combined with the uniform dispersion of NiFe alloy within the carbon matrix, synergistically optimized impedance matching conditions. This facilitated the penetration of incident EMWs into the material and enabled multiple reflections and scatterings of EMWs within the internal spatial framework of NFNCAs, thereby enhancing EMW attenuation. Secondly, the conduction loss resulted from the activation of conductive carriers that migrated along the conductive network of NFNCAs under alternating electromagnetic field excitation. This process finally transformed EMW energy into thermal energy [74]. Thirdly, the structural defects originating from residual functional groups and nitrogen doping within the carbon matrix served as dipole centers under the influence of an electromagnetic field, thus inducing dipole polarization [75–77]. The numerous heterogeneous interfaces formed between NiFe alloy nanoparticle cores and carbon shells facilitated charge accumulation, leading to interfacial polarization [78–80]. Finally, magnetic loss contributions from NiFe alloy nanoparticles enhanced the overall attenuation performance. Thus, the synergistic integration of these multiple attenuation mechanisms enabled NFNCAs to exhibit exceptional EMW absorption performance.

### Real-Time Monitoring of Structural Integrity

**Fig. 5:**
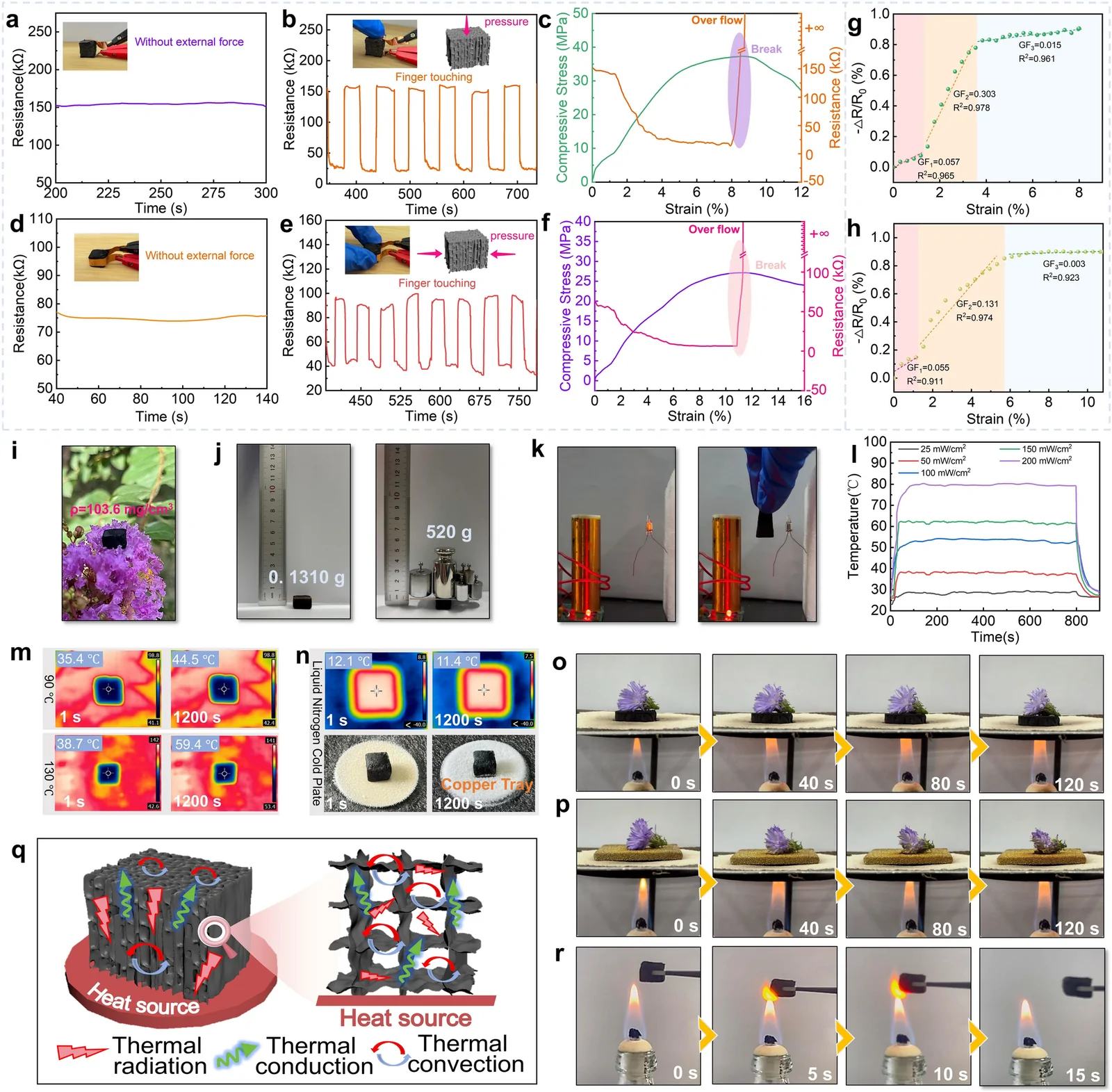
Multifunctional characteristics of NFNCAs. Resistance-time curves of NFNCA-2 in the longitudinal direction: **a** No external applied force, and **b** slow pressing and releasing with fingers. **c** Stress–strain curves and corresponding resistance changes of NFNCA-2 during compression in the longitudinal direction. Resistance-time curves of NFNCA-2 in the transverse direction: **d** No external applied force, and **e** slow pressing and releasing with fingers. **f** Stress–strain curves and corresponding resistance changes of NFNCA-2 during compression in the transverse direction. *GF* values of NFNCA-2 during compression in the **g** longitudinal and **h** transverse directions. **i** Photograph of NFNCA-2 on the petals. **j** Compression test and **k** Tesla coil test of NFNCA-2. **l** Photothermal temperature curves of NFNCA-2 under different light power densities. **m** Infrared thermal images of NFNCA-2 on 90 °C and 130 °C thermostatic heating plates. **n** Infrared thermal images and optical photographs of NFNCA-2 on the cold plate in liquid nitrogen (-196 °C) at different times. **o, p** Thermal insulation performance of NFNCA-2. **q** Schematic diagram of the thermal insulation mechanism of NFNCA-2.** r** Flame retardancy of NFNCA-2

Interestingly, the 3D cross-linked conductive network of NFNCA could serve as strain sensors to detect changes in the internal structure of NFNCA. As demonstrated in Fig. 5a, the longitudinal resistance of NFNCA-2 remained constant without external force applied. When pressure was slowly applied and released with fingers, the electrical signal decreased or increased accordingly (Fig. 5b). Real-time electrical signal measurement enabled the detection of NFNCA plastic deformation and structural damage. As the compressive strength of NFNCA-2 gradually increased, its internal resistance gradually decreased due to the enhanced contact between conductive carbon skeletons. When the applied stress reached the material’s ultimate strength, the resistance mutated and exceeded the threshold (Fig. 5c). This was because exceeding the stress limit triggered catastrophic structural failure, and the resulting collapse of the NFNCA skeleton severed the interconnected conductive pathways, leading to an open-circuit-like state and a sharp rise in resistance. Similar piezoresistive behavior was observed in the transverse direction (Fig. 5d-f).

The gauge factor (*GF*, Eq. S19), defined as the rate of relative resistance change to applied strain, is commonly used to characterize the strain sensitivity of a sensor. The curves of Δ*R*/*R*_0_ versus compressive strain demonstrated that the sensitivity of NFNCA-2 could be divided into three parts in the longitudinal direction (Fig. 5g). For the strain range of 0-1.3%, the *GF* was maintained at a low level of 0.049. This phenomenon stemmed from the formation of the limited number of discrete contact points during the initial deformation stage, thus leading to low electron transport efficiency. Within the strain range of 1.3%-3.6%, the *GF* value rose markedly to 0.303. This enhanced sensitivity stemmed from the reduction in the distance between the conductive skeletons and the alteration of contact interfaces under compression, thereby effectively regulating the contact resistance and enhancing the electron transmission efficiency. However, as the strain exceeded 3.6%, the *GF* value decreased to 0.015. This decrease could be ascribed to the transformation of the contact mechanism. The initial point contact converted to surface contact, which restricted structural deformation, hindered the formation of new conductive pathways, and thus suppressed electron hopping and transport. Likewise, the sensitivity behavior with three distinct ranges was observed in the transverse direction (Fig. 5h). The *GF* value in the longitudinal direction was higher than that in the transverse direction. This might be due to the fact that the unidirectional structure could create effective conductive pathways along the continuous honeycomb walls in the longitudinal direction, thereby resulting in a higher relative resistance change rate under the same strain [81].

Based on the above results, the internal structure of NFNCA could be monitored by observing the resistance changes, thus realizing real-time monitoring of its structural integrity in operation. This inherent self-sensing capability is highly promising for enhancing the reliability of NFNCA, making it particularly valuable for high-stakes applications such as a lightweight filler in aircraft wings or protective casings for sensitive electronics. In these scenarios, the ability to promptly detect micro-cracks or impact damage in situ and without the need for disassembly is critical for ensuring mission success and preventing catastrophic structural failure.

### Multifunctional Properties

The porous NFNCA-2 exhibited a low density (~ 103.6 mg cm^−3^) and was sufficiently light, enabling it to stand steadily on the flower petals (Fig. 5i). Moreover, NFNCA-2 could support a load approximately 4000 times its own weight without suffering mechanical damage, demonstrating its excellent mechanical properties (Fig. 5j). The combination of lightweight and robust load ability positioned this novel absorbing material as a prime candidate for structural–functional integrated absorber, with significant potential in sectors such as portable electronics and aerospace. Furthermore, the suppression effect of NFNCA-2 on EMW propagation was verified through two practical application experiments. In the first experiment, an electromagnetic field generated by the Tesla coil formed an electromotive force to light the bulb when switching on the power. However, the bulb went off when placing NFNCA-2 between the coil and the bulb (Fig. 5k). In the second experiment, during the Bluetooth communication test of two smartphones, placing NFNCA-2 over the hole in the tinfoil resulted in signal transmission blockage (Fig. S18, detail in Supporting Information). These phenomena confirmed that NFNCA-2 could provide effective EMW absorption capability for electronic devices.

The porous surface of NFNCA-2 was conducive to multiple reflections and absorption of incident light, and it exhibited excellent photothermal conversion capability. Under simulated solar irradiation (xenon lamp), the photothermal conversion of porous NFNCA-2 was evaluated. Specifically, the equilibrium temperatures of NFNCA-2 were 29.1, 38.7, 54.1, 62.7, and 80.4 °C, respectively, at light power densities of 25, 50, 100, 150, and 200 mW cm^−2^ (Fig. 5l). After switching off the lamp, NFNCA-2 cooled down rapidly to ambient temperature, indicating an efficient and reversible photothermal response. Overall, these findings confirmed the effective thermal management capability of NFNCA and highlighted its considerable potential for applications in electronic devices.

To satisfy the escalating requirements for applications in demanding environments, advanced EMWA materials need to possess multiple functions such as thermal insulation and flame retardancy. The EMWA materials with excellent thermal insulation performance can effectively protect electronic devices from damage caused by overheating or supercooling temperatures [82]. The thermal insulation performance of NFNCA was evaluated using an infrared thermal imager. NFNCA-2 was positioned on two heating stages set at 90 and 130 °C. The material’s surface temperature showed a slow upward trend within 20 min (Fig. S19), while stabilizing at 44.5 and 59.4 °C, respectively (Fig. 5m), demonstrating its outstanding thermal insulation performance. In addition, when placed on a copper tray frozen in liquid nitrogen, NFNCA-2 maintained a consistent temperature (11.4 °C) at the top even after 20 min (Figs. 5n and S19). Furthermore, with the extension of freezing time, a thick frost layer formed on the surface of the copper tray beneath the aerogel, while the NFNCA-2 surface was not frozen. Moreover, the area of the palm not covered by NFNCA-2 displayed a different color from the surrounding area and could be clearly distinguished under infrared detection equipment (Fig. S20). In contrast, the area of the palm covered by NFNCA-2 showed the same color as the surrounding area and became invisible under infrared detection equipment. Overall, the remarkable thermal insulation performance endowed NFNCA-2 with effective shield infrared thermal radiation, indicating that it has promising potential in infrared stealth applications.

To further investigate the thermal insulation capability of NFNCA, the flower was placed on the wire gauze insulated with NFNCA-2 (5 mm thickness), followed by heating of the wire gauze using an alcohol lamp. The results demonstrated that after 120 s, both the shape of the flower and the NFNCA-2 were kept almost unchanged (Fig. 5o). For comparison, a thermally conductive copper foam with the same thickness as NFNCA-2 was employed as the control sample and placed between the flower and the heated wire gauze as a thermal conductive medium under the identical heating condition. In this case, the flower was observed to be charred and shrunk into a small piece after 120 s (Fig. 5p). These results revealed that NFNCA had excellent thermal insulation performance, as evidenced by its low thermal conductivity of 0.056 W m^−1^ K^−1^, along with excellent high-temperature resistance, thus making it a promising candidate for applications in harsh thermal environments. The excellent thermal insulation performance of NFNCA-2 could be attributed to the following reasons. On the one hand, the internal high porosity of NFNCA-2 could reduce the solid-phase thermal conductivity and radiative heat transfer efficiency. On the other hand, the porous structure greatly extended the heat conduction path, and decreased the heat transfer capacity (Fig. 5q) [83].

The flame retardancy of NFNCA was evaluated through alcohol lamp combustion, microscale combustion calorimeter (MCC), and limiting oxygen index (LOI) tests. Notably, NFNCA-2 didn’t ignite, with only localized shrinkage and deformation observed at the flame-contact surface in an alcohol lamp for 15 s (Fig. 5r). Furthermore, the MCC test showed that when NFNCA-2 was heated to 850 °C in a N_2_/O_2_ mixed atmosphere, both the peak heat release rate (PHRR) and heat release capacity (HRC) were detected as zero, and the oxygen consumption during the combustion process was extremely low (only 0.6%) (Table S3). This proved that NFNCA-2 hardly participated in gas-phase combustion reactions, indicating it had excellent flame retardancy and the ability to inhibit combustion spread. Meanwhile, the MCC data revealed that even after a continuous heating process lasting up to 2200 s, the residual carbon content of NFNCA-2 remained as high as 83.8 wt%. The corresponding 16.2 wt% mass loss was attributed to the slight thermal decomposition of NFNCA-2 in a high-temperature air atmosphere. In addition, the LOI was also measured to quantitatively assess flame retardancy. The LOI value of NFNCA-2 was 46.3%, which further confirmed its outstanding flame retardancy. The excellent flame retardancy of NFNCA-2 could be ascribed to the synergistic effect of multiple mechanisms. Firstly, the N-doped carbon matrix could release non-flammable gases (N_2_, NH_3_) to dilute oxygen and flammable components in the gas phase. Secondly, embedded NiFe nanoparticles could work as catalysts to promote the formation of a dense and stable char layer on the surface of NFNCA-2, which could provide a good physical barrier. Thirdly, the hierarchical porous structure could also dissipate heat and avoid heat accumulation in the material [84–86]. On the whole, the combination of excellent thermal insulation and flame retardancy makes NFNCA more promising for applications in harsh environments.

This work demonstrated a 3D hierarchical biomimetic honeycomb-like porous magnetic NFNCA that integrated radar-infrared stealth, piezoresistive sensing, thermal management, and flame retardancy. Compared with similar reported carbon aerogels [87–98], the advantage of NFNCA-2 lay not in leading every single metric (Table S4) but in achieving the synergistic integration of multiple key functionalities. It retained excellent wave absorption performance, while simultaneously exhibiting outstanding thermal insulation, mechanical stability, flame retardancy, and the real-time structural integrity monitoring capability. Thus, NFNCA represented a more balanced and integrated solution, and its comprehensive multifunctional performance made it far more practically valuable for scenarios requiring synergistic multi-capability integration. The synergistic integrated performance originated from the distinct components (NiFe nanoparticles, N-doped carbon matrix) and honeycomb-like 3D conductive network structure, which were constructed via a rationally designed fabrication strategy combining in situ growth, unidirectional freeze-drying, and pyrolytic carbonization. Overall, this study provides a feasible and efficient strategy to design high-performance carbon-based aerogels for diverse practical applications.

## Conclusions

In summary, we fabricated biomimetic honeycomb-like porous magnetic NFNCA with low density, high dispersion of magnetic nanoparticles, strong interfacial connectivity, and multifunctionality via a straightforward strategy involving in situ growth, freeze-drying, and pyrolysis carbonization. The rational component design and pore structure of NFNCA resulted in efficient EMWA performance. Benefiting from integrated radar-infrared stealth capabilities, reliable thermal management performance, and superior flame retardancy, NFNCA was an ideal candidate for more complicated and varied environments. Specifically, NFNCA-2 maintained an EAB of 6.24 GHz at 1.60 mm and achieved a minimum *R*_L_ value of −53.49 dB as well as a specific *R*_L_ value of −138.58 dB mm^−1^ at 1.93 mm. COMSOL simulations confirmed the effectiveness of NFNCA in reducing radar scattering intensity and its potential for practical applications. Interestingly, the 3D cross-linked conductive network of NFNCA-2 with a high graphitization degree could serve as strain sensors to monitor the structural integrity of materials in real-time, thus avoiding potential safety hazards. Furthermore, the rapid photothermal conversion of NFNCA-2 confirmed its excellent thermal management ability. Meanwhile, the outstanding thermal insulation capability and flame retardancy of NFNCA-2 enabled its practical application in high-temperature conditions. With high performance, facile fabrication, and multifunctionality, NFNCA holds broad and promising application prospects in fields such as electromagnetic compatibility and protection, electronic devices, thermal management, real-time self-sensing structural health monitoring, and aerospace.

## Supplementary Information

Below is the link to the electronic supplementary material.

Supplementary file1 (DOCX 13605 KB)
