# Supplementary material for: Self-Sensing NiFe@N-doped Carbon Aerogel: Integrating Excellent Radar Stealth, Inherent Structural Health Monitoring, Thermal Management, and Flame Retardancy
Source: Nanomicro Lett. 2026 Mar 10;18:278. doi: 10.1007/s40820-026-02128-5 (PMC12976249; doi:10.1007/s40820-026-02128-5)
Supplement: Supplementary file 1 — Supplementary file1 (DOCX 13605 KB) [file 40820_2026_2128_MOESM1_ESM.docx]

Supporting Information for

**Self-Sensing NiFe@N-doped Carbon Aerogel: Integrating Excellent Radar Stealth, Inherent Structural Health Monitoring, Thermal Management, and Flame Retardancy**

Xiaosen Du^1, 3^, Jianhua Zhou^1, 3,^*, Jiarui Yu^1, 3^, Xiaoyan Nie^1, 3^, Mingyu Luo^1, 3^, Xingyuan He^1, 3^, Anguo Xiao^2^

^1^ College of Bioresources Chemical and Materials Engineering, Shaanxi University of Science and Technology, Xi’an 710021, P. R. China

^2^ Hunan Provincial Key Laboratory of Water Treatment Functional Materials, Hunan University of Arts and Science, Changde 415000, P. R. China.

^3^ National Demonstration Center for Experimental Light Chemistry Engineering Education (Shaanxi University of Science and Technology), Xi’an 710021, P. R. China

*Corresponding author. E-mail: [zhoujianh@21cn.com](mailto:zhoujianh@21cn.com) (Jianhua Zhou)

S1 Experimental Section

**S1.1 Materials**

Cellulose nanofibrils (CNFs) dispersion with 6 wt% solid content was provided by Langfang City Shandian Technology Co., Ltd. (Langfang, China). Potassium hexacyanoferrate(III) (K_3_Fe(CN)_6_), trisodium citrate dihydrate (Na_3_C_6_H_5_O_7_·2H₂O), nickel nitrate hexahydrate (Ni(NO_3_)_2_·6H_2_O), and sodium hydroxide (NaOH) were bought from Sichuan Xiling Science Co., Ltd. (Chengdu, China). Sodium periodate (NaIO_4_), sodium acetate, anhydrous ethanol, ethylene glycol, acetic acid, and hydroxylamine hydrochloride were bought from Aladdin Biochemical Technology Co., Ltd. (Shanghai, China). All chemicals were of analytical grade (AR) and used without further purification. The water was ultrapure water. Collagen was prepared from waste gray skin scraps according to the literature [S1, S2]. The thermally conductive copper foam (50 × 50 × 5 mm) was purchased from Kunshan Jin’anjia New Material Co., Ltd. (Jiangsu, China).

**S1.2 Fabrication of DCNF**

DCNF was prepared according to the method reported earlier with modifications as described [S3]. Firstly, 1.2833 g of sodium periodate, 1.0017 g of CNF (dry weight), and 25 mL of acetic acid buffer solution (pH=3.5) were mixed, followed by the addition of deionized water to a final volume of 200 mL. The mixture was stirred at 45 ℃ without light for 48 h. Then 20 mL of ethylene glycol was added to remove the residual oxidant. The obtained solution was poured into excess anhydrous ethanol to obtain a precipitate, which was washed alternately with water and ethanol several times to completely remove iodic compounds. Finally, the DCNF washed to neutrality was freeze-dried and stored in the dark.

**S1.3 Determination of DCNF Aldehyde Content**

The aldehyde content of DCNF was determined through hydroxylamine hydrochloride/sodium hydroxide method [S4]. Principles of the reaction are as following:

-CHO + NH_2_OH·HCl → -CHNOH + HCl + H_2_O

NaOH + HCl → NaCl + H_2_O

In detail, the DCNF suspension solution (0.1 g DCNF in 30 mL deionized water) was adjusted to pH 4.5 with HCl and then mixed with a hydroxylamine hydrochloride solution (2 g hydroxylamine hydrochloride in 20 mL deionized water, pH=4.5). The mixture was magnetically stirred at room temperature for 24 h and subsequently titrated to pH 4.5 with 30 mM NaOH solution. The aldehyde content was determined via Equation (S1).

$W=\frac{C_{NaOH}\times V_{NaOH}}{m}$ (S1)

where *W* is aldehyde content (mmol/g), *C*_NaOH_ denotes the concentration of sodium hydroxide solution (mol/L), *V*_NaOH_ represents the volume of NaOH solution used (mL), and *m* is the weight of DCNF (g).

**S1.4 Characterization**

A field emission scanning electron microscope (SEM, S4800, Hitachi, Japan) was used to examine the samples' microscopic morphology and elemental distribution. A transmission electron microscope (TEM, Tecnai F20, FEI, Netherlands) was utilized for the analysis of the samples’ lattices. The FTIR spectra of the samples were obtained using a FTIR spectrometer (EQUINOX55, Bruker, Germany). A powder X-ray diffractometer (XRD, D8ADVANCE A25, Bruker, Germany) with Cu Kα radiation (λ=0.154 nm) was used to determine the molecular structures of the materials. A Raman spectroscope (Lab RAM, DXRxi, ThermFisher, USA) was used to examine the degree of graphitization between 500 and 2500 cm^-1^ while being excited by a 532 nm laser. The elemental states of the samples were determined by an X-ray photoelectron spectroscope (XPS, ESCALAB 250Xi, TFS, USA) using an aluminium Kα-ray source. A double station physical adsorption instrument (ASAP 2460, Micromeritics, USA) was used to determine the specific surface area and pore size distribution. At room temperature, a vibrating sample magnetometer (VSM, 7404, Lakeshaw, USA) was used to evaluate the magnetic properties of the samples. An infrared thermal camera (FLIR E4, USA) was used to measure the temperature variations of the sample under heating and cooling conditions, thereby reflecting changes in thermal insulation performance of the sample. The resistances of NFNCAs were measured by a digital multimeter (DMM 6500, Keithley, USA), and the conductivity was calculated by equation (*σ*=*H*/*RA*, where H, *R*, and *A* are the height, resistance, and cross-sectional area of the test samples, respectively.). The thermal conductivity of NFNCA-2 was measured by a thermal constant analyzer (TPS-3500, Hot Disk, Sweden). The flame retardancy test was conducted using a microscale combustion calorimeter (MCC, PX01008, Jiangsu Ferman Security Technology Co., Ltd., China). The limiting oxygen index (LOI) value was evaluated with an oxygen index instrument (ZR-01, Qingdao Ruixinjie Instrument Co., Ltd., China). A vector network analyzer (VAN, PNA-N5244 A, Agilent, USA) was used to test the electromagnetic parameters of each sample in the frequency range of 2-18 GHz by coaxial method, and then the EMWA characteristics of the samples were further evaluated. The 20 wt% sample and 80 wt% molten paraffin (mass fraction) were completely mixed and then pressed to form a ring with an inner diameter of 3.04 mm and an outer diameter of 7.00 mm.

**S1.5 RCS** **Simulation**

Using computer simulation technology (CST; studio Suite 2020), we simulated the radar cross section (RCS) of NFNCAs in the actual far-field environment. The simulation model was a 20 cm × 20 cm double-layer plate construction. The model’s top was a 1.93 mm thick absorbing layer, which was an absorbent coating composed of NFNCAs and molten paraffin, while the bottom was a 1.00 mm thick perfect electric conductor (PEC). The NFNCA/PEC model plate was placed on the *X-O-Y* plane, and linear polarized plane EMW was incident from the positive direction of the *Z*-axis to the negative direction of the *Z*-axis. Meanwhile, the *X*-axis was the path taken by electric polarization as it spread. The incident azimuth angles were restricted within the range of -90° to 90°. 11.44 GHz was selected as the field monitor frequency, with the open boundary conditions specified in all directions. The RCS values could be determined using the Equation (S2) [S5].

$\sigma\left( dB m^{2} \right)=10log{(\frac{4\pi S}{\lambda^{2}}\left| \frac{E_{s}}{E_{i}} \right|)}^{2}$ (S2)

where *S* is the area of the established model, *λ* represents the wavelength of EMW, *E_s_* is the electric field intensity of scattered EMW and *E_i_* is the electric field intensity of incident EMW.

**S1.6 COMSOL Simulation**

The COMSOL Multiphysics was employed to model and simulate the distribution of electric field intensity, magnetic field intensity, surface current density and power loss density of NFNCA. The coating model measured 10 mm in length, 10 mm in width, and 1.93 mm in thickness.

**S2 Supplementary Eq****uations**

According to the Maxwell-Garnett theory, the effective permittivity (*ε*_eff_) of a material consisting of two different components could be expressed by the following equations:

$\varepsilon_{eff}^{MG}=\varepsilon_{1}\frac{\left( \varepsilon_{2}+2\varepsilon_{1} \right)+2\rho\left( \varepsilon_{2}-\varepsilon_{1} \right)}{\left( \varepsilon_{2}+2\varepsilon_{1} \right)-\rho\left( \varepsilon_{2}-\varepsilon_{1} \right)}$ (S3)

where *ε*_1_ and *ε*_2_ are the permittivity of host and guest components, respectively, *ρ* is the volume fraction of the guest. In this work, the host and guest components represented the tight NiFe/carbon and the air. Thus, the *ε*_2_ was equal to 1.

$\varepsilon_{eff}^{MG}=\varepsilon_{1}\frac{\left( 1+2\varepsilon_{1} \right)+2\rho\left( 1-\varepsilon_{1} \right)}{\left( 1+2\varepsilon_{1} \right)-\rho\left( 1-\varepsilon_{1} \right)}<\varepsilon_{1}$ (S4)

Therefore, the porous structures could reduce the effective permittivity.

The magnetic loss originated from the interactions between magnetic domains or dipoles with alternating magnetic fields. In the high frequency region, the magnetic loss mainly consisted of domain wall rotation, eddy current loss, ferromagnetic resonance including natural resonance and exchange resonance. The natural resonance frequency could be calculated by the following equations:

$2\pi f_{r}=\gamma H_{\alpha}$ (S5)

$H_{\alpha}=4\left| K_{1} \right|/3\mu_{0}Ms$ (S6)

$K_{1}=\mu_{0}MsHc/2$ (S7)

where *H_α_* is the anisotropic energy, *γ* is the gyroscopic ratio, *f_r_* is the resonant frequency, *μ_0_* is the permeability of free space and *K_1_* is the anisotropy.

Based on the metallic backing model and transmission line theories, the reflection loss (*R*_L_) in this work was calculated by electromagnetic parameters. The calculation equations were shown as follows:

$R_{L}=20lg\left| \frac{Z_{in}-Z_{0}}{Z_{in}+Z_{0}} \right|$ (S8)

$Z_{in}=Z_{0}\sqrt{\frac{\mu_{r}}{\varepsilon_{r}}}tanh\left( j\frac{2\pi fd\sqrt{\mu_{r}\varepsilon_{r}}}{c} \right)$ (S9)

where *Z_in_, Z_0_, f, d, μ_r_, ε_r_*, *c* and *j* denote the input impedance, free space impedance, frequency, matched thickness, relative complex permeability, relative complex permittivity, velocity of EMW in free space, and the imaginary unit respectively.

The EMW absorption efficiency (*η_abs_*) was calculated by the following equation:

$\eta_{abs}\left( \% \right)=100-\left( \frac{1}{{10}^{\frac{\left| R_{L} \right|}{10}}} \right)\times100$ (S10)

In the quarter-wavelength matching model, the theoretical matching thickness (*t*_m_) could be calculated by the following equation:

$t_{m}=\frac{n\lambda}{4}=\frac{nc}{4f_{m}\sqrt{\left| \mu_{r} \right|\left| \varepsilon_{r} \right|}}\left( n=1, 3, 5\ldots\right)$ (S11)

where *t_m_* is the matching thickness, *f_m_* is the matching frequency corresponding to the minimum *R*_L_ value, and λ is the propagation velocity.

The internal polarization process of materials could be shown using Cole-Cole curves. To elucidate the dielectric loss mechanism, the Debye dipole relaxation process was defined by the following equation:

$\left( \varepsilon^{'}-\frac{\varepsilon_{s}+\varepsilon_{\infty}}{2} \right)^{2}+\left( \varepsilon^{''} \right)^{2}=\left( \frac{\varepsilon_{s}-\varepsilon_{\infty}}{2} \right)^{2}$ (S12)

where *ε_s_* is the static permittivity and *ε*_∞_ is the relative permittivity at infinite frequency.

The linear dependence of *ε′* on *ε′′*/*f* was adopted to identify the dielectric loss type. The specific equation was as follows:

$\varepsilon^{''}=\frac{\omega\tau(\varepsilon_{s}-\varepsilon_{\infty})}{1+\omega^{2}\tau^{2}}+\frac{\sigma}{{2\omega\varepsilon}_{0}}$ (S13)

The conduction loss (*ε_c_''*) and polarization loss (*ε_p_''*) were calculated by the following equation:

$\varepsilon^{''}\left( \omega\right)=\varepsilon_{p}^{''}+\varepsilon_{c}^{''}=\left( \varepsilon_{s}-\varepsilon_{\infty} \right)\frac{\omega\tau}{1+\omega^{2}\tau^{2}}+\frac{\sigma}{\varepsilon_{0}\omega}$ (S14)

where *σ* is the electrical conductivity, *τ* is relaxation time, *ε*_0_ is the vacuum dielectric constant (8.85×10^-12^ F/m), and *ω* is the angular frequency (*ω*=2π*f*). Therefore, *ε"* can be divided into two parts: *ε_p_"* caused by relaxation (or polarization) and *ε_c_"* caused by electrical conductivity.

Under the motivation of alternating magnetic fields, the induced current turning around the conductor was called eddy current, which transformed magnetic field energy into heat. The presence of eddy current could be judged by *C*_0_ value, which was calculated by the following equation:

$C_{0}=\mu^{''}\left( \mu^{'} \right)^{-2}f^{-1}=2\pi\delta d^{2}\mu_{0}$ (S15)

where *μ'* and *μ''* are the real part and imaginary part of permeability, *μ*_0_ is the initial permeability, *f* is the frequency, *σ* is the electrical conductivity, *d* is the thickness.

The impedance matching (*Z*) was calculated by the following equation:

$Z=\left| \frac{Z_{in}}{Z_{0}} \right|=\left| \sqrt{\frac{\mu_{r}}{\varepsilon_{r}}tanh\left[ \frac{2\pi jfd}{c}\sqrt{\mu_{r}\varepsilon_{r}} \right]} \right|$ (S16)

The attenuation constant (*α*) was calculated by the following equation:

$\alpha=\sqrt{2}\pi f/c\sqrt{\left( \mu^{''}\varepsilon^{''}-\mu^{'}\varepsilon^{'} \right)+\sqrt{\left( \mu^{''}\varepsilon^{''}-\mu^{'}\varepsilon^{'} \right)^{2}+\left( \mu^{''}\varepsilon^{'}+\mu^{'}\varepsilon^{''} \right)^{2}}}$ (S17)

The specific *R*_L_ (*SR*_L_) value was used to explain the minimum *R*_L_ value *vs* thickness and filler rate, and evaluated the material’s comprehensive advantages in terms of light weight, thin thickness, and efficient absorption properties. The calculation equation was shown as follows:

$SR_{L}=\frac{MR_{L}}{d_{m}\times f}$ (S18)

where the *MR*_L_ represents the minimum *R*_L_ value, *d*_m_ is the matching thickness at the *minimum* *R*_L_, and *f* is the filling rate of the absorbing material.

The gauge factor (*GF*) was calculated by the following equation:

$GF=\frac{\triangle R}{R_{0}\varepsilon}$ (S19)

where Δ*R* is the resistance changing with compressive strain, *R*_0_ is the resistance before strain, and *ε* is the applied strain.

**S3 Supplementary Figures**

**Fig. S1** **a** Effect of DCNF content on the density and porosity of NFNCAs. **b-c** Compression stress-strain curves of NFNCAs with different DCNF contents

In this experiment, DCNF rich in aldehyde groups was used to cross-link collagen protofibrils, and the effect of DCNF content (calculated relative to the mass of collagen protofibrils) on the density, porosity, and mechanical properties of NFNCAs was investigated. As shown in Fig. S1**a**, with increasing DCNF content, the density of NFNCAs gradually increased and the porosity decreased progressively. This was attributed to the Schiff base reaction between the aldehyde groups on DCNF and the amino groups in collagen protofibrils. As the DCNF content increased, the number of Schiff base bonds gradually rose, thereby enhancing the cross-linking density. This drove the structural transition of NFNCAs from a loose configuration to a more compact porous network structure, accompanied by a progressive increase in density and a corresponding reduction in porosity. In addition, the incorporation of the cross-linking agent DCNF significantly improved the mechanical properties of NFNCAs. Figs. S1**b** and S1**c** show the stress-strain curves of NFNCAs with different DCNF contents. It was observed that compressive strength first increased as the DCNF content increased from 0 to 45 wt%, and then decreased as the DCNF content further increased. When the DCNF content was 45 wt%, the compressive strength showed the maximum value (37.34 MPa). This was because as the DCNF content increased, the number of Schiff base bonds rose, which in turn elevated the cross-linking density. This effect strengthened the rigidity and structural stability of the carbon skeleton, thereby significantly enhancing the mechanical properties of NFNCAs. However, when the DCNF content exceeded 45 wt%, the compressive strength of NFNCAs decreased significantly. This phenomenon was attributed to excessive cross-linking bonds that rendered the 3D network structure of the carbon aerogel excessively dense, making it prone to brittle fracture under external stress and thereby resulting in a reduction in compressive strength. Therefore, 45 wt% was selected as the optimal DCNF content.

**Fig.** **S2** FTIR spectra of CNF, DCNF, collagen protofibril, and NFPA-0

**Fig. S3 a** FTIR spectra of Ni-PBA particles and NFPAs, **b** Local magnification of the hydroxyl group peaks in FTIR spectra

**Fig. S4** Zeta potentials of collagen-DCNF dispersions (precursors of NFPA-0, NFPA-1, NFPA-2, and NFPA-3) with Ni-PBA loading content


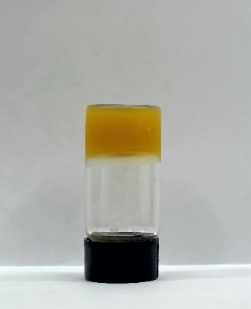


**Fig. S5** Photograph of the jelly-like NFPA precursor

**Fig. S6** Electrical conductivity of NFNCAs with NiFe-PBA loading content

**Fig. S7** XPS survey spectra of NFNCA-0: **a** full spectrum, **b** C 1s spectrum, and **c** O 1s spectrum

**Fig. S8** XPS survey spectra of NFNCA-2: **a** full spectrum, **b** O 1s spectrum

**Fig. S9** **a-c** EMWA performance of NFNCA-0

**Fig. S10 a-b** EMWA performance of NFNCA-1

**Fig. S11 a-b** EMWA performance of NFNCA-2

**Fig. S12 a-b** EMWA performance of NFNCA-3


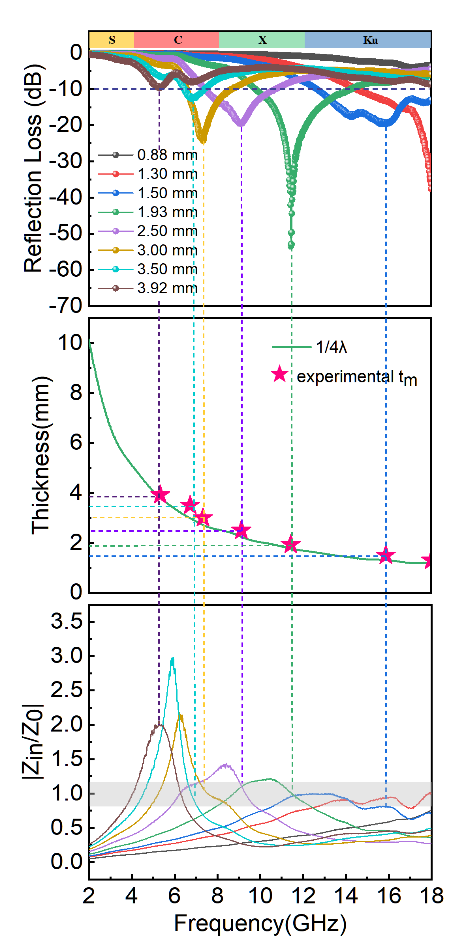


**Fig. S13** *R*_L_ curves, calculated matching thickness, experimental matching thickness, and impedance matching (Z) of NFNCA-2

**Fig. S14 a** Tangent value of dielectric constant and **b** tangent value of permeability of NFNCAs

**Fig. S15** Plots of *ε_c_"* and *ε_p_"* versus frequency: **a** NFNCA-0, **b** NFNCA-1, **c** NFNCA-2, and **d** NFNCA-3

**Fig. S16** Two-dimensional patterns of impedance values of **a** NFNCA-0, **b** NFNCA-1, and **c** NFNCA-3

**Fig. S17** Attenuation constant of NFNCAs


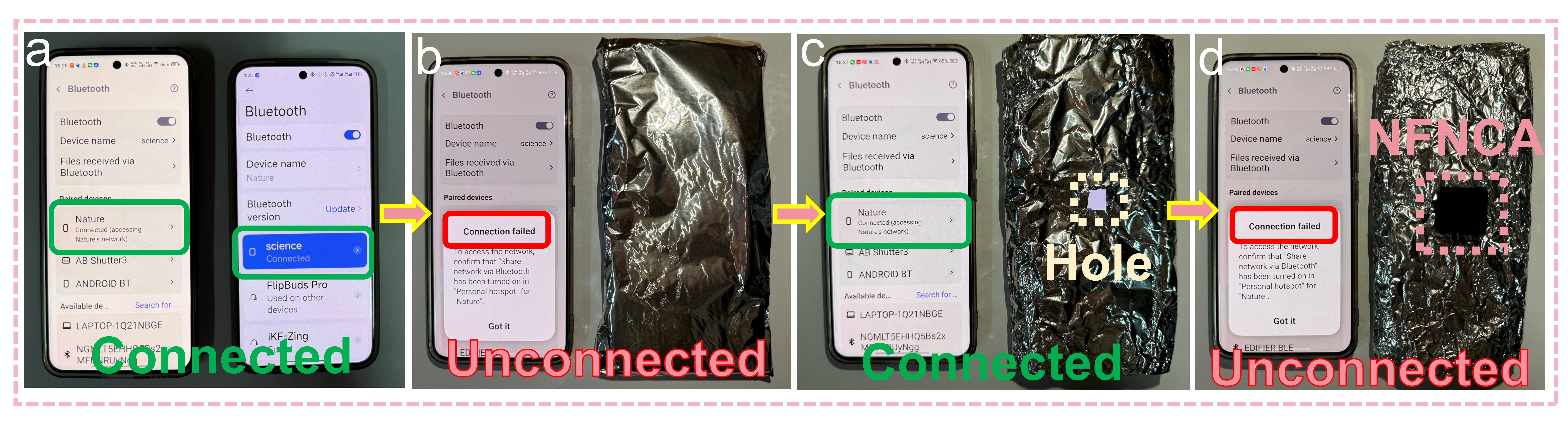


**Fig. S18** Photographs of the Bluetooth connection between two smartphones

To further verify the interaction between NFNCA-2 and EMWs, an evaluation was performed with two smartphones serving as the test devices. When two smartphones were set to Bluetooth pairing mode and placed within the effective communication range, a stable wireless Bluetooth connection was successfully established between them (Fig. S18a). In contrast, when one smartphone was enclosed in tinfoil, the connection was immediately disrupted due to the EMW blocking by the tinfoil (Fig. S18b). Subsequently, the two smartphones were found to reconnect with each other when opening a hole in the tinfoil (Fig. S18c). However, covering this hole with NFNCA-2 interrupted the Bluetooth connection (Fig. S18d). These phenomena demonstrated that NFNCA-2 could provide effective EMW absorption for electronic devices.

**Fig. S19** Surface temperature change curves of NFNCA-2 on the hot plate (90 ℃ and 130 ℃) and liquid nitrogen (-196 ℃) cold plate


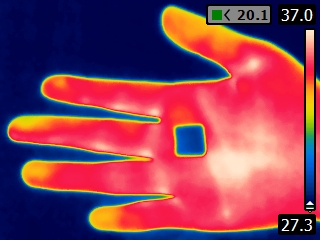


**Fig.** **S20** Infrared thermal image of NFNCA-2 placed on the palm

**S4 Supplementary Tables**

**Table S1** Relative mass content of various nitrogen species in NFNCAs

| Samples | Pyridinic N (wt%) | Pyrrolic N (wt%) | Graphitic N (wt%) |
| --- | --- | --- | --- |
| NFNCA-0 | 46.51 | 53.49 | / |
| NFNCA-2 | 51.56 | 33.57 | 14.87 |

**Table S2** Comparison of thickness, minimum *R*_L_, EAB, filler rate, and specific *R*_L_ of the NFNCA-2 with the other microwave absorption materials

| Samples | EAB (GHz) | Minimum *R*_L_ (dB) | t_EAB_ (mm) | t*_R_*_L_ (mm) | Filler rate (wt%) | Specific *R*_L_ | Refs. |
| --- | --- | --- | --- | --- | --- | --- | --- |
| Fe_3_O_4_/Co-Ni polyhedrons | 6.40 | -46.60 | 1.90 | 1.90 | 20 | -122.63 | [48] |
| Fe-Ni/C nanorods | 2.00 | -30.00 | 6.13 | 2.00 | 15 | -100.00 | [49] |
| Fe@Ni(40) | 1.89 | -41.63 | 3.10 | 2.90 | 25 | -57.42 | [50] |
| Fe_3_O_4_-Ni-C | 4.50 | -59.40 | 2.40 | 2.40 | 50 | -49.50 | [51] |
| SiCN/Fe/Ni | 4.21 | -52.06 | 1.43 | 1.54 | 30 | -112.68 | [52] |
| Fe_20_Ni_80_ composites | 5.40 | -39.50 | 2.20 | 3.00 | 15 | -87.78 | [53] |
| FeCoNi/CMC/  MXene aerogels | 5.44 | -27.29 | 1.42 | 4.61 | 50 | -11.84 | [54] |
| Sugarcane carbon aerogel | 5.80 | -44.89 | 4.30 | 4.30 | 10 | -104.40 | [55] |
| MOF/Aerogel/  CFF | 5.20 | -53.20 | 2.20 | 2.20 | 30 | -80.61 | [56] |
| CoNi-CAs | 5.70 | -40.69 | 1.76 | 2.41 | 30 | -56.28 | [57] |
| Joint carbon aerogel | 1.71 | -27.27 | 8.00 | 8.00 | 10 | -34.09 | [58] |
| SiC@PDA composite aerogel | 6.24 | -24.36 | 2.00 | 2.50 | 10 | -97.44 | [59] |
| C/Co aerogel | 4.52 | -52.50 | 1.40 | 1.40 | 30 | -125.00 | [60] |
| CoNi@graphene aerogel | 4.20 | -42.62 | 1.50 | 1.50 | 26 | -109.28 | [61] |
| Co-N/C@graphene hybrid aerogel | 5.30 | -46.80 | 2.10 | 2.70 | 20 | -86.67 | [62] |
| Co_x_Ni_y_/carbon hybrid aerogel | 4.05 | -62.09 | 1.65 | 4.15 | 25 | -59.85 | [63] |
| NFNCA-2 | 6.24 | -53.49 | 1.60 | 1.93 | 20 | -138.58 | This work |

Note: The specific information of Refs. [48-63] can be found in the References section of the manuscript.

**Table S3** MCC test data of NFNCA-2

| Sample | PHRR (W/g) | HRC (J/(g·K)) | Oxygen consumption(%) | Char residue  (wt%) |
| --- | --- | --- | --- | --- |
| NFNCA-2 | 0 | 0 | 0.6 | 83.8 |

**Table S4** Comparison of multifunctional performances and fabrication strategy of NFNCA-2 with other microwave absorption materials

| Samples | Density (mg/cm^3^) | Minimum *R*_L_ (dB) | EAB (GHz) | Load-bearing capacity (times self-weight) | Compressive strength (MPa) | Thermal conductivity (W⋅m^-1^⋅K^-1^) | Flame retardancy | Sensing performance | Fabrication strategy | Ref. |
| --- | --- | --- | --- | --- | --- | --- | --- | --- | --- | --- |
| CoMo-LDO@Co/C aerogel | 139.10 | -52.74 | 7.93 | / | 4.39 | 0.068 | Alcohol lamp flame retardancy | / | Non-directional freeze-drying | [87] |
| Ti₃C₂Tₓ MXene@CoFe-MOF@chitosan carbon aerogel | 35.09 | -50.95 | 6.10 | 500 | / | / | Alcohol lamp flame retardancy | / | Non-directional freeze-drying | [88] |
| MXene/C | 50.00 | -53.02 | 5.30 | / | / | / | / | Piezoresistive sensing | Non-directional freeze-drying | [89] |
| CNTs/PI composite aerogels | 45.00 | -41.00 | 6.88 | 500 | / | 0.061 | / | / | Unidirectional freeze-drying | [90] |
| ZnO/SiC_nw_ aerogel | 167.00 | -42.40 | 4.20 | 1786 | 34.97 | 0.056 | / | / | Unidirectional freeze-drying | [91] |
| Zirconia fibers reinforced carbon aerogel | 164.00 | -80.30 | 5.16 | 2000 | / | 0.034 | / | / | Ambient pressure random drying | [92] |
| SiBCN/SiBCN_nf_ composite aerogel | 105.00 | -20.00 | 5.60 | / | 1.62 | / | / | / | Non-directional freeze-drying | [93] |
| MoC_x_ hybrid polyimide aerogel | 23.00 | -47.72 | 4.58 | 2000 | / | / | Alcohol lamp flame retardancy | / | Unidirectional freeze-drying | [94] |
| Co@NC aerogel | 38.61 | -40.49 | 5.36 | 800 | / | / | / | / | Non-directional freeze-drying | [95] |
| MXene/carbon fiber/polyimide composite aerogel | 18.58 | -61.96 | 3.97 | / | 1.685 | / | Alcohol lamp flame retardancy | / | Non-directional freeze-drying | [96] |
| C/SiOC composite aerogel | 248.00 | -67.85 | 6.88 | 1700 | / | 0.067 | Alcohol lamp flame retardancy | / | Ambient pressure random drying | [97] |
| C/SiOC aerogel | 402.00 | -65.45 | 4.90 | 3700 | / | 0.081 | Alcohol lamp flame retardancy | / | Ambient pressure random drying | [98] |
| NFNCA-2 | 103.60 | -53.49 | 6.24 | 4000 | 37.34 | 0.056 | Alcohol lamp flame retardancy; LOI=46.3% | Piezoresistive sensing | Unidirectional freeze-drying | This work |

Note: The specific information of Refs. [87-98] can be found in the References section of the manuscript.

**Supplementary References**

1. Y. Z. Meng, P. F. Weng, Extraction of collagen by pepsin from the cattle skin. Fine Chemicals **27**(12), 1187-1279 (2010). <https://doi.org/10.13550/j.jxhg.2010.12.021>
2. Z. H. Tian, X. Li, H. Wang, The properties of collagen extracted from pickled skin. Journal of Shaanxi University of Science & Technology **37**(2), 17-23 (2019). <https://doi.org/10.19481/j.cnki.issn2096-398x.2019.02.004>
3. Y. Zhang, L. Zhang, S. Shao, X. Chang, M. Li, Periodate oxidation of carboxymethyl cellulose under controlled conditions. ChemistrySelect **5**(22), 6765–6773 (2020). <https://doi.org/10.1002/slct.202000470>
4. L. Dai, Y. Geng, X. Ding, Z. Zhang, C. Lai et al., Highly stretchable, self-adhesive, and biocompatible cellulose/chitosan based double network hydrogel for wound dressing. Carbohydr. Polym. **366**, 123869 (2025). <https://doi.org/10.1016/j.carbpol.2025.123869>
5. W. Gu, S.J.H. Ong, Y. Shen, W. Guo, Y. Fang et al., A lightweight, elastic, and thermally insulating stealth foam with high infrared-radar compatibility. Adv. Sci. **9**(35), 2204165 (2022). <https://doi.org/10.1002/advs.202204165>
